# Microfilament-coordinated adhesion dynamics drives single cell migration and shapes whole tissues

**DOI:** 10.12688/f1000research.10356.1

**Published:** 2017-02-17

**Authors:** Rocio Aguilar-Cuenca, Clara Llorente-Gonzalez, Carlos Vicente, Miguel Vicente-Manzanares

**Affiliations:** 1 Universidad Autonoma de Madrid School of Medicine, Madrid, Spain; 2 Instituto de Investigacion Sanitaria Hospital Universitario de la Princesa, Madrid, Spain; 3 Team of Cell Interactions in Plant Symbiosis, Faculty of Biology, Complutense University, Madrid, Spain

**Keywords:** adhesion, migration, integrin, actin, myosin, II, clutch, morphogenesis

## Abstract

Cell adhesion to the substratum and/or other cells is a crucial step of cell migration. While essential in the case of solitary migrating cells (for example, immune cells), it becomes particularly important in collective cell migration, in which cells maintain contact with their neighbors while moving directionally. Adhesive coordination is paramount in physiological contexts (for example, during organogenesis) but also in pathology (for example, tumor metastasis). In this review, we address the need for a coordinated regulation of cell-cell and cell-matrix adhesions during collective cell migration. We emphasize the role of the actin cytoskeleton as an intracellular integrator of cadherin- and integrin-based adhesions and the emerging role of mechanics in the maintenance, reinforcement, and turnover of adhesive contacts. Recent advances in understanding the mechanical regulation of several components of cadherin and integrin adhesions allow us to revisit the adhesive clutch hypothesis that controls the degree of adhesive engagement during protrusion. Finally, we provide a brief overview of the major impact of these discoveries when using more physiological three-dimensional models of single and collective cell migration.

## Introduction

Adhesion is a fundamental cellular property that shapes the architecture of complex, multicellular tissues and enables their maintenance. Adhesion also participates in the navigation of individually migrating cells (for example, immune cells) across tissues. In multicellular tissues, cells interact stably with other cells as well as with the extracellular matrix (ECM). Individually migrating cells also interact with other cells as well as with the matrix, although these interactions tend to be transient.

Research carried out during the last 40 years has yielded a detailed molecular understanding of the molecular components of cell-cell and cell-matrix adhesions. This includes molecular receptors and adaptors, their stoichiometry, and the dynamics of their interactions. Also, recent efforts have highlighted the role of adhesion as a point of functional convergence of biochemical and biophysical signals. Adhesion is controlled by the interplay of adhesive receptors and their cytoplasmic binding partners with the cellular cytoskeleton, which controls the size and dynamics of adhesive contacts. In turn, adhesion shapes the architecture of the cell. The objective of this review is not to provide a comprehensive revision of the molecular players involved in different types of adhesive interactions, since many excellent reviews predate the present work. Instead, we aim to integrate old and new insights to describe the dynamics of the adhesive events that shape cells and tissues along a biophysically and biochemically controlled timeline. These control mechanisms determine the duration and strength of the adhesive process as well as its downstream effects in the biology of cells and tissues.

## Specific multimolecular adhesive complexes mediate specialized cellular tasks

Multicellular organisms have acquired intricate levels of molecular complexity to perform different functions at specialized anatomic locations. Evolution has enabled multicellular organisms to develop organs specialized in coordination and awareness (nervous system), defense against pathogens (immune system), energy and oxygen capture (digestive and respiratory systems, respectively), and so on. These diverse scenarios require uniquely shaped organs based on different cell-cell and cell-matrix adhesive events to form specific architectures that adapt best to each function. For example, some cell-cell contacts need to last for years (for example, between cognitive neurons) ^1^. Other types of cell-cell contacts are required to endure mechanical stress (for example, muscle or blood vessels). Yet other contacts are, by definition, transient and short-lived (for example, the interaction of immune cells with the endothelial lining of the blood vessels during extravasation). These diverse adhesive interactions require the formation of different types of molecular complexes, including at least one type of adhesion receptor and a variable number of adaptors. Over the years, many original articles and reviews have provided some clarity on the participation of many of these molecules. Although many membrane receptors bear adhesive potential (for example, carbohydrate-based receptors, tight junction proteins, and many others), two major families of adhesive receptors are involved in the maintenance of structural integrity of the tissues and also participate in single-cell migration: cadherins and integrins.

- Cadherins: Cadherins mediate stable cell-cell adhesion between cells. They do so mostly in a homophilic fashion; that is, cadherins of the same type expressed by two different cells interact in a *trans* fashion ^2, 3^. Heterotypic contacts between different cadherins have also been reported (reviewed in 4).
- Integrins: Integrins are heterodimeric receptors that have cellular as well as extracellular ligands; thus, they can support cell-cell as well as cell-matrix interactions. Many different classifications have been established, mainly depending on the molecular composition of the heterodimers or the nature of their ligands ^5^. From a functional standpoint, integrins can also be divided into the following:
  - Constitutively active integrins, which hold cells together in tissues.
  - Inducible integrins, which mediate transient interactions between cells with other cells or with matrices but only when the physiological scenario requires it, for example during inflammation or blood clotting.

Receptors are the most important part of adhesive molecular complexes. However, they require extra components to integrate the function of adhesion into the global cellular response to its microenvironment. Each type of receptor recruits a collection of specific cytoplasmic and plasma membrane components that control the nature of the signals transmitted to the rest of the cell. These molecules include regulators of the activity of the receptor; intermolecular adaptors; enzymes; cytoskeletal components and linkers; and many others. Their binding sites and availability dictate the stoichiometry and dynamics of the adhesion as well as the functional adaptations and responses of the cell to the adhesive event.

Perhaps the most common example of the role of adhesion in the control of global cellular outcomes is the morphological adaptation of the cell to the microenvironment. Adhesive contacts trigger intracellular signals that promote cytoskeletal remodeling, reshaping the cell. A typical outcome is a cytoskeletal-dependent reinforcement of the adhesive contact. In this process, an initial adhesive signal triggers the reorganization of the cytoskeleton at the cellular region involved in the adhesive contact. Cytoskeletal reorganization typically recruits additional receptors or intermolecular adaptors or both, increasing the area of adhesive contact. However, contact reinforcement is hardly the only outcome of the establishment of adhesive molecular complexes. Different types of signals emanate from adhesive contacts, providing positional and contextual information to a given cell, potentially directing its proliferation, migration, differentiation, and so on. In this light, adhesive regions become focal signaling points. Perhaps this is why the term “focal adhesion” ^6^, initially referred to as “adhesion plaque” ^7^, is an apt and prescient definition that has remained in use for over 40 years to designate integrin-based, cell-matrix adhesive areas.

When the dynamics of adhesive contacts are analyzed according to the functional classification established above, several trends emerge:

1. It is predictable that stable adhesive contacts have a rapid growth phase and a very slow disassembly phase, although this has not been tested directly. During the growth phase, adaptor signaling enables the growth of the adhesive region to a certain threshold size, which is defined by the abundance of ligand and its affinity for the receptor as well as the existence of specific intracellular cues (for example, the shape of underlying cytoskeletal patterns formed in response to the adhesion).
2. Transient adhesive structures display rapid growth but equally rapid turnover. In general, adhesive molecular complexes displaying receptors bearing low affinity for their ligands mediate transient contacts, even when the receptors face a great abundance of ligand. However, transient interactions are not exclusive to low-affinity receptors, and constitutively active receptors may mediate this behavior too ^8^. In both cases, adaptor signaling is designed to trigger additional responses or enhance the release of the adhesive contact or both (reviewed in 9).
3. In general, stable and long-lived adhesive contacts require intricate and sophisticated cytoskeletal backbones, whereas transient contacts are less stringent.
4. Though obvious, the point needs to be underscored that strong adhesion counters rapid cell motility and supports high structural persistence, and vice versa; weak adhesion supports cell migration but transient structural stability.

In the following sections, we will focus on specific aspects of different adhesive molecular complexes in steady state and in pathology and describe in more detail some aspects of the general points outlined in this section.

## Let’s stay together: cadherin-based cell-cell adhesions

Cadherins are the lynchpin of cell-cell adhesion in epithelial tissues ^10^. They are transmembrane proteins bearing extracellular Ig-like domains and intracellular short domains that selectively recruit cytoplasmic adaptors, particularly catenins. Cadherins display tissue-selective isoform specificity, which has been reviewed elsewhere ^11, 12^. Cadherins mainly interact with other cadherins through homophilic (same cadherin) interactions of the extracellular domains. Cadherins of different types may also form heterophilic bonds. Through their intracellular domains, cadherins interact with adaptors of the catenin family, particularly β- and γ-catenin (plakoglobin). β-catenin and plakoglobin recruit α-catenin, which is an actin-binding protein that connects cadherin complexes to microfilaments, but only under tension ^13^; its affinity for actin filaments is very low *in vitro* (that is, under no tension, when complexed with E-cadherin and β-catenin) ^14^. In fact, cadherin-based complexes act as mechanosensors that react to the application of mechanical strain. Tension across α-catenin while bound to β-catenin on one end and actin on the other enables the conformational extension of α-catenin ^3^. This change triggers the reinforcement of the contact by amplifying its interaction with microfilaments. A major mediator of such amplification step is the actin adaptor vinculin. Strained α-catenin recruits vinculin to the cell-cell adhesion, which increases the actin-binding capability of the entire complex ^15, 16^. Importantly, vinculin recruitment to cadherin-based adhesions is governed by phosphorylation ^17^.

**Figure 1.**
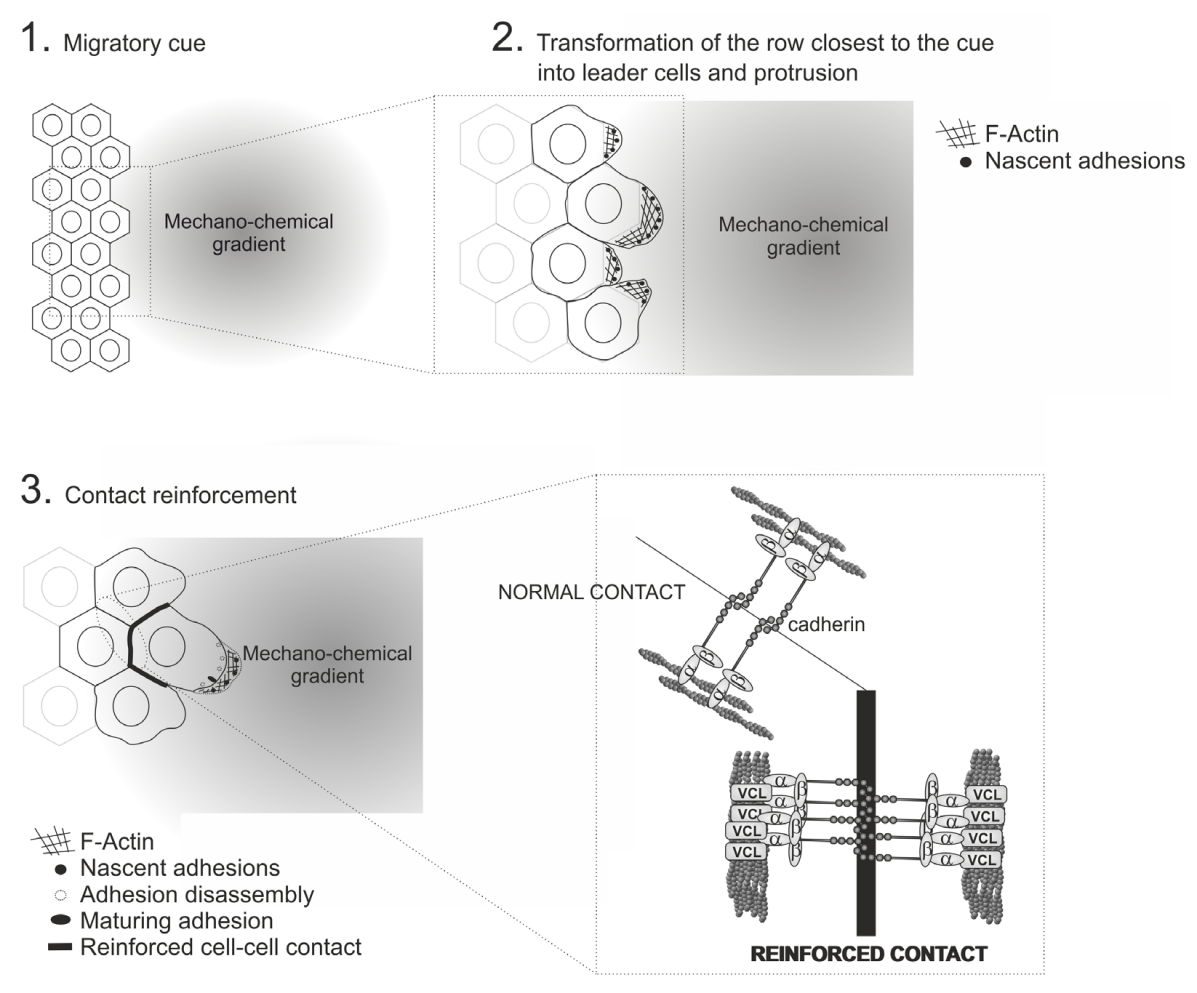
Effect of the emergence of a migratory cue next to an epithelial monolayer. Panels depict the morphological and dynamic changes that follow the emergence of a mechanical or chemical migratory cue in the form of a gradient next to an epithelial monolayer. ( **1**) The row of cells closer to the cue become leader cells. ( **2**) Leader cells develop migratory traits, characterized by the emergence of actin-rich protrusions in the direction of the cue. Integrin-based nascent adhesions assemble underneath lamellipodial protrusions to stabilize the migratory edges. ( **3**) Continued protrusion causes the emergence of newly formed nascent adhesions, the disassembly of most nascent adhesions of the previous generation at the end of the lamellipodium, and the stabilization of a few of them. In addition, the forward motion of the leader cell applies strain to the cell-cell contacts at the rear and rear sides. Bottom right insert represents contact reinforcement caused by mechanical strain. Strain applied to the cadherin-β-catenin-α-catenin complex causes the conformational extension of α-catenin, which recruits additional actin adaptors (for example, vinculin, or VCL). This results in an amplification of actin and cadherin recruitment.

Multiple reviews have highlighted the molecular composition, stoichiometry, and dynamics of cadherin-based cell-cell adhesions ^2, 3, 10, 11^. Their purpose is to maintain the integrity of tissues made of continuums of cells interacting laterally (for example, epithelial tissues). Cadherins also maintain the coherence of cell monolayers during morphogenetic events or homeostatic processes (for example, wound healing) that involve the migration of entire cohorts of epithelial cells. In this manner, the sequence of events (as shown schematically in Figure 1) would be as follows:

1. In response to a migratory cue, which can be mechanical, chemical, or a combination of the two, the cells closer to the origin of the cue start moving toward the center of the gradient.
2. The movement of these “leader” cells applies mechanical strain to the cadherin-dependent adhesions that connect them to the first row of “follower” cells.
3. Cadherin-containing mechanosensing complexes perceive the strain and reinforce the contact to counterbalance it, compensating the increased force per area by increasing the number of binding sites (recruitment of additional cadherin receptors, which lowers the stress per bond) or by dissipating the work through an increased number of interacting meshwork filaments (F-actin).

This simple model of strain rerouting/dissipation bears important predictions:

1. Strain is transmitted from the extracellular medium to the intracellular medium through F-actin.
2. Strain dissipation will increase rapidly as additional receptors/actin filaments are recruited to the focal adhesive point, which means that the back rows of the monolayer will be subject to much less strain than the front rows (that is, those closer to the leader cells and the migratory cue). In most cases, owing to proliferation-based extension of the monolayer, this is not critical.
3. Local mechanisms must exist to convert mechanical strain into chemical signals to increase receptor binding, promote actin recruitment or both.

These predictions have been demonstrated in different models. Leader cells influence the shape and behavior of follower cells ^18^. Cadherin signaling, alterations in the overall packaging of the epithelial structure, and mechanotransduction events drive the communication between leader and follower cells ^19, 20^. Other studies have highlighted the progressive decay of force transmission across epithelial monolayers ^21, 22^, which is consistent with strain dissipation through contacts bearing increased cadherin receptors, enhanced connections to actin or both. Finally, mechanisms to convert strain into an altered recruitment capability are only beginning to be understood. Several studies have highlighted the interplay between chemical signaling and mechanical forces, the control of collective cell migration and the maintenance of cell-cell contacts during this process ^23, 24^. Also, force application to α-catenin stabilizes its binding to vinculin ^25^, enhancing its linkage to the actin cytoskeleton and improving strain dissipation or transmission to neighbor cells or both. Cadherin complexes also mediate actin polymerization to increase F-actin accumulation at cell-cell contacts and maintain monolayer integrity ^26^. Another recent study shows that cadherin adhesions accumulate at the rear of leader cells in collective migrating endothelial cells, serving as guidance cues to direct the migratory responses of follower cells ^27^. As the molecular complexity of cadherin-based complexes emerges, additional mechanically active regulatory elements will be unveiled.

An interesting pathologic scenario that illustrates this issue is the connection of cancer-associated fibroblasts (CAFs) to tumor cells. Different studies have shown how CAFs are required for the efficient dissemination of tumor cells ^18^. But how are CAFs and tumor cells connected? CAFs are mesenchymal cells; that is, they do not express E-, but N-cadherin ^20^, which can also interact with β-catenin and thus recruit α-catenin/actin to the receptor complex. On the other hand, tumor cells do not downregulate E-cadherin expression during the initial stages of dissemination. Hence, it is possible that CAFs establish heterotypic N-cadherin/E-cadherin connections with tumor epithelial cells. Though different in composition from the complexes described above, N-cadherin/E-cadherin heterophilic bonds are due to respond to strain application in a similar manner to homophilic E-cadherin/E-cadherin interactions, reinforcing the contacts between CAFs and tumor cells as the former migrate outside the tumor, driving tumor cells with them. Another non-excluding possibility is that cadherin-integrin cross-talk at adherens junctions (integrins are also present in these regions) could regulate these processes ^28^.

## The clutch players in your team: integrin-based cell-matrix adhesions

Cell-matrix adhesions provide traction for cell movement and enable the correct shaping of multicellular tissues. This is a crucial event in development and regenerative schemes. Integrins mediate most stable interactions of cells with different types of extracellular matrices. Unlike cadherin-based contacts, cell-matrix adhesions turn over frequently and form again to permit cell movement when a migratory requirement emerges. This is controlled through the conformational activation of integrins. Integrins are able to persist in a low-affinity conformation even in the presence of a large amount of their ligand. Conversely, intracellular recruitment of specific adaptors to the tail of integrins (for example, talin) promotes inside-out integrin activation even in the presence of small amounts of ligand. In this manner, integrins are tunable machines that can be dialed up (“stickier”) or down (“less sticky”). Importantly, inside-out integrin activation critically depends of the integrin type: for example, platelet (α _IIB_β _3_) and leukocyte (α _L_β _2_ and α _4_β _1_) integrins need to be tunable since alterations to their activation, or inhibition, would result in catastrophic scenarios for the homeostasis of an entire organism. Conversely, integrins that function in tissue contexts (for example, fibroblast or epithelial integrins) are activated by default to enable immediate, efficient, and persistent adhesion to the matrix. Importantly, in some tissue-specific contexts, integrins can form very long-lasting interactions (for example, myotendinous junctions and muscle costameres) (reviewed in 29, 30). Although no quantitative information exists regarding specific compositional differences between transient cell-matrix integrin-based adhesions and stable contacts such as those specified above, it is predictable that their molecular makeup determines their turnover. This may include the presence of muscle-specific molecules (for example, meta-vinculin and dystrophins), which could influence the integrin-actin linkage, determining the duration of the contact.

In addition, integrins transmit many types of signals from the extracellular medium to the cell, enabling survival and proliferation, instructing the cells to migrate or differentiate, and so on. Integrins act as anchorage points for large multimolecular complexes that include hundreds of different molecules with varied association/dissociation kinetics and stoichiometry. This concept alone endows integrin-based adhesions with great regulatory flexibility. Several excellent studies and reviews have dealt with the molecular complexity of integrin-based adhesions ^31, 32^. Here, we focus on their ability to reshape cells and tissues through its connection to the actin cytoskeleton.

### Integrin-actin linkages

**Figure 2.**
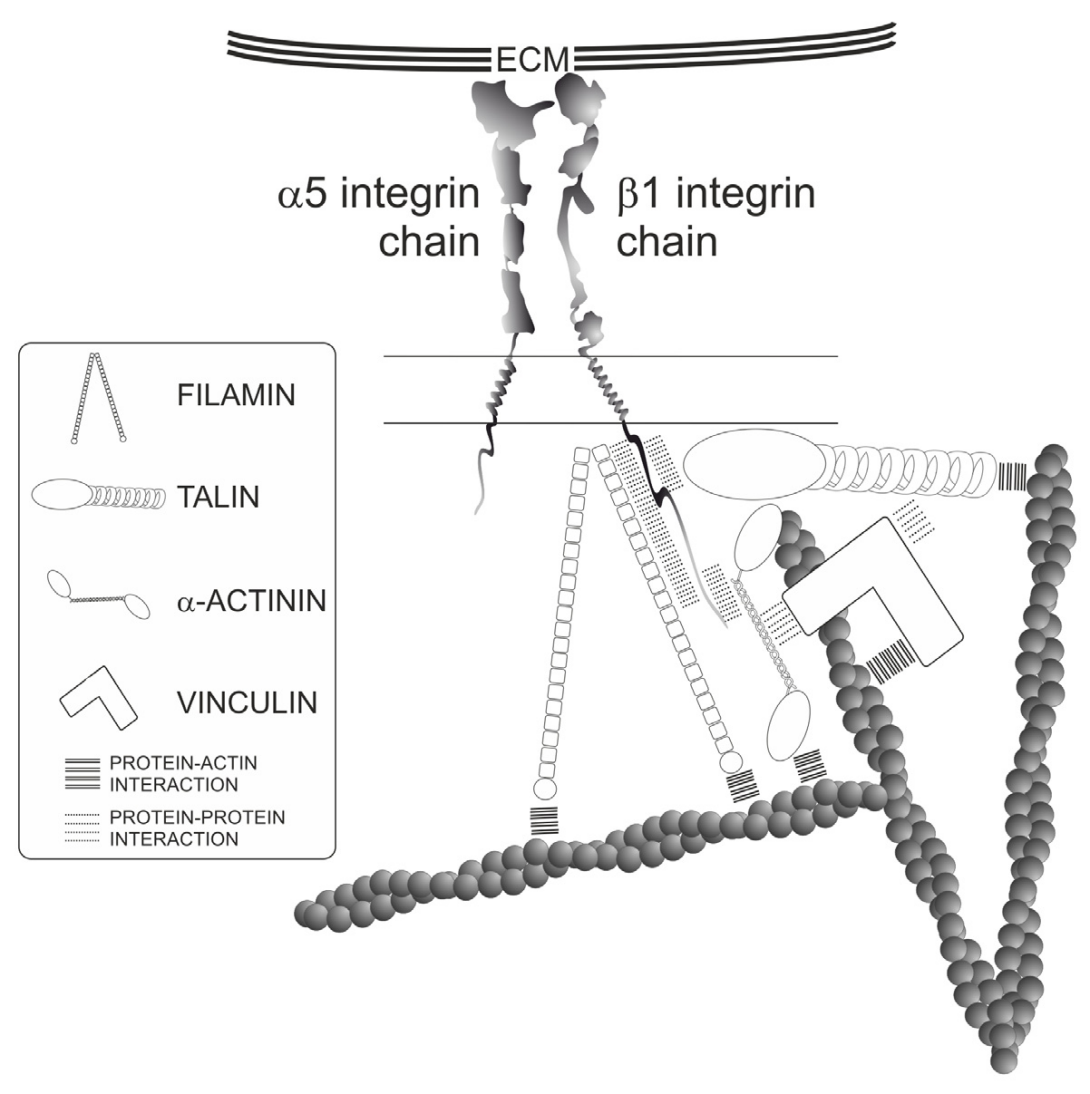
Key integrin-actin linkages. Cartoon depicts the connections of α _5_β _1_ integrin with actin. Upon integrin-ligand interaction, the tail of β _1_ integrin interacts with filamin through most of its C-terminus cytoplasmic domain, with α-actinin through distal segment of the cytoplasmic domain and with talin through the membrane proximal segment of the intracellular tail. Talin and α-actinin also interact with vinculin. Integrin-adaptor and adaptor-adaptor interactions are represented as dashed lines; adaptor-actin interactions as solid lines. Filamin cross-links actin filaments orthogonally, whereas α-actinin cross-links filaments in various ways, including parallel and anti-parallel modes. Vinculin and talin are primarily adhesion-actin linkers. ECM, extracellular matrix.

Different molecules potentially link integrin-based adhesions to the actin cytoskeleton ( Figure 2). Some of them can bind directly to integrins through one domain and F-actin through another (for example, filamin ^33^, α-actinin ^34^, and talin). Importantly, these molecules undergo accumulation at stress sites, which could facilitate adhesion growth ( 35 and see below). In addition to their direct role as integrin-actin linkers, they can modulate the role of other adaptors in this process; for example, filamin A is an inhibitor of integrin function that is recruited to integrins in the absence of talin ^36^. However, the best-characterized linker of this type is talin, which is a centerpiece of integrin conformational activation ^37^. Application of molecular strain to talin promotes a conformational change that enables its interaction with integrins through its N-terminus and actin filaments through its C-terminus. In addition, talin binds to vinculin in a strain-dependent manner ^38^, thereby multiplying the anchorage of the entire complex to actin. Another regulator, Kank2, controls the strength of the interaction between talin and integrin, thereby controlling cellular adhesiveness and migratory speed ^39^. These connectors, together with other adaptors and enzymes of the adhesive interactome, constitute the core of a regulatory mechanism that controls the degree of engagement of integrins and actin and the ECM. This molecular complex is a tunable clutch-like mechanism that is ultimately responsible for the functional flexibility of integrin-based adhesions, which ranges from long-term immobilization on the ECM to fast, gliding cellular motility.

### Dissecting the adhesive molecular clutch

**Figure 3.**
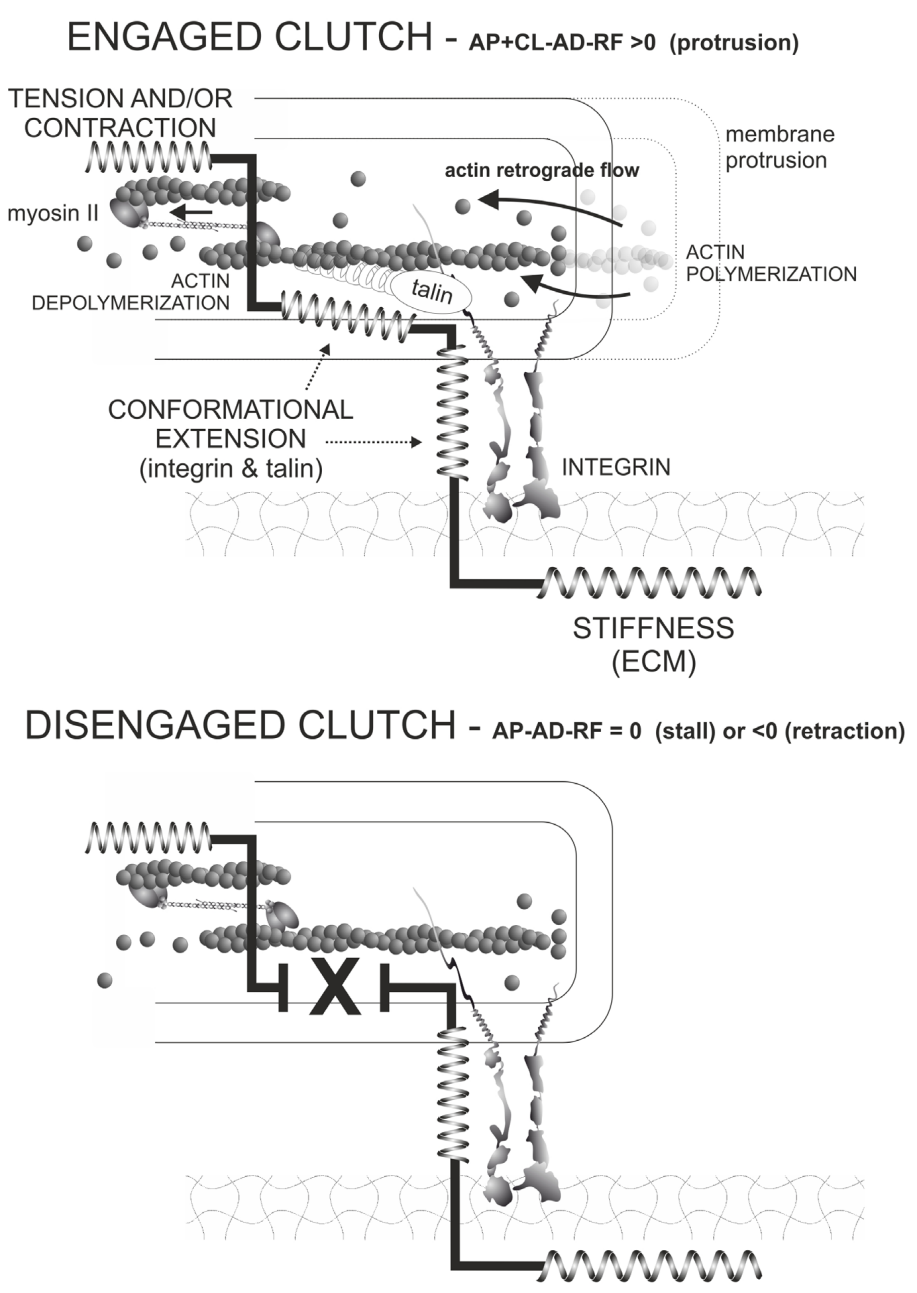
The adhesive molecular clutch. Schematic representation of the engaged (top) and disengaged (bottom) clutch. Mechanically active modules—extracellular matrix (ECM), integrin, talin, and myosin II—are represented by spring designs. Note the molecular continuum formed by mechanically active elements when the clutch is engaged, which opposes the stalling/retracting effect of retrograde flow (RF) and the collapsing effect of actin disassembly (AD) at the rear of the protrusion. Arrows at the front denote RF and, under myosin II, indicate contractile force or tension. A simple numerical notation is used, in which the balance of actin polymerization (AP), AD, the adhesive clutch (CL), and RF can be larger than zero (protrusion), equal to zero (stall), and lower than zero (retraction). The bottom part of the figure represents one of the possible causes of clutch disengagement, in this case talin disappearance, and the concomitant loss of mechanical connection among the parts of the clutch. Other inhibitory treatments also block clutch engagement (for example, integrin blockade, lower ECM stiffness, or increased myosin II activation).

The adhesive molecular clutch hypothesis was initially formulated to explain the relationship between adhesion and actin-based protrusion in neuronal growth cones ^40^. A simplified explanation of the inner workings of this adhesive clutch is as follows: membrane protrusion (that is, forward advancement) is driven by actin polymerization as long as the growing actin filaments are anchored to an adhesive point that remains immobile (“engaged” clutch) ( Figure 3). Newly polymerized actin pushes the membrane forward, creating membrane tension and triggering retrograde flow of material in this region. If the filaments are not anchored, polymerization at the barbed end causes no membrane protrusion because it is compensated by retrograde flow (“disengaged” clutch). In this view, polymerization on a disengaged clutch is futile: the growth of the filament only maintains the position of the tip because of the backwards drag of the entire filament.

Whereas the essence of the molecular clutch hypothesis is as stated above, the discovery of multiple connectors of variable stoichiometry and dynamics that link integrins to actin draws a very complex, multilayered regulatory scenario that critically depends on the integrin subtype that mediates the adhesive contact. It also extends the concept of the molecular clutch from the engagement or disengagement of actin retrograde flow with nascent adhesions in the lamellipodium (as observed in growth cones) to the engagement or disengagement of actin stress fibers with mature focal adhesions in slow-moving cells (for example, fibroblasts).

Presumably, constitutively active integrins (for example, α _5_β _1_) will engage the adhesive clutch more efficiently, generating strong myosin II-dependent forces ^41^. Adhesive stability and myosin II activation drive the subsequent emergence of complex actin structures associated with the adhesions. In addition, the long actin bundles associated with growing adhesions promote the recruitment of additional integrins to adhesive sites along the actin template, which increases the size of the adhesive contact and locally enhances adhesiveness (avidity). This indicates that strong clutch engagement or high myosin II activation or a high number of activated receptors (or a combination of these) would create adhesive resistance to protrusion ^42, 43^. Conversely, weak clutch engagement or low myosin II activation or low numbers of ligated receptors (or a combination of these) would promote protrusiveness. Interestingly, these conditions do not necessarily translate into faster migration, which fits a Gaussian behavior instead ^44^. The reason for this apparent contradiction likely lies in the integration of the phenomena that add up to enable cell migration. Indeed, cells with a low degree of clutch engagement, or myosin II activation, protrude faster ^42, 45, 46^. However, myosin II inhibition results in a deficient retraction of the rear ^42, 47^ and nuclear repositioning ^48^, and an altered balance between adhesion formation and turnover ^8^, which would explain deficient cellular migration despite intense protrusion. Conversely, inducible integrins (for example, α _L_β _2_) are unable to generate the growth of adhesion-associated actin bundles, resulting in negligible adhesion growth. Interestingly, these integrins trigger very robust myosin II activation ^46^ but seem not to invest it in an adhesive clutch mechanism but in localizing it to the rear ^49^ probably to push the nucleus, which is a major steric hindrance for movement ^50^.

The mechanics of the substrate also govern clutch engagement ( Figure 3). The rigidity of the substrate tunes up or down integrin activity by controlling the efficiency of the transmission of the traction generated inside cells to the ECM. On soft substrates, integrin engagement is low, and myosin II-dependent force transmission is poor, allowing adhesive slippage ^51^. As a result, the clutch is mainly disengaged, which causes ineffective protrusion. On stiff substrates, myosin II-generated forces are better transmitted, the clutch is engaged, and protrusion occurs as described above. Not surprisingly, talin is a key integrator of substrate rigidity. Talin-deficient cells cannot transform substrate rigidity into cellular traction. In these cells, higher rigidities do not translate in a better engagement to actin, and myosin II-dependent forces are either not generated or not transmitted efficiently to the substratum, resulting in loss of protrusiveness ^52^. Similar to talin, other adhesive adaptors are also mechanoreactive (for example, integrin receptors themselves ^53^ and p130CAS (BCAR1)). BCAR1 contains a cryptic Src site (Tyr165), which is accessible only when the molecule is stretched mechanically ^54^. The role of BCAR1 in clutch engagement is less obvious as it does not bind actin directly, although it could regulate this process through secondary interactions.

The clutch model predicts the existence of a tunable connection between a mechanically flexible substrate and a force-generating device (myosin II) through a series of conformationally deformable connectors (for example, integrins and talin; see above). When the clutch is engaged, it contributes to actin polymerization overcoming the resistance against forward motion caused by retrograde flow, resulting in protrusion. Conversely, when the clutch is disengaged, actin polymerization is balanced, or surpassed, by depolymerization plus the retrograde flow, resulting in stall or retraction ( Figure 3).

It is important to note that the strong dependence of the adhesive molecular clutch for epithelial and mesenchymal cells to migrate likely reflects an adaptation of these cells to move in response to a migratory cue. These cells need to interact strongly with their surroundings, and the adhesive clutch turns this interaction in a multimolecular mechanism that enables protrusion and migration. Conversely, the apparent lack of a strong adhesive clutch in fast, individually migrating cells (for example, leukocytes) suggests that these cells have reduced their dependence of the microenvironment in order to migrate quickly and efficiently.

### The adhesive clutch integrates force and biochemical signals to enable actin-propelled membrane protrusion

The adhesive clutch is controlled biochemically by the linkage of the integrin receptors to the actin filaments. It is also controlled mechanically by the rigidity of the extracellular microenvironment. But how are these parameters conjoined to explain the protrusiveness of migrating cells? A key integrator is myosin II ^55^. Myosin II converts biochemical energy (ATP hydrolysis) into mechanical force. Forces generated by the ATP-dependent conformational movement of the myosin II head domain are propagated through actomyosin cables. In this manner, centripetal traction at adhesive foci is generated by myosin II and translated into the substratum through the adaptor proteins that form the adhesive clutch, including the integrin receptor itself. Myosin II is dispensable for the assembly of nascent adhesions and the strong retrograde flow observed at the lamellipodium, which defines approximately the first micrometer of the protruding edge of the cell ^8, 56^. Conversely, myosin II inhibition prevents adhesion maturation in rigid, two-dimensional (2D) substrates ^8^ and greatly decreases the retrograde flow of actin in the lamellum (that is, the region posterior to the lamellipodium (1–10 μm away from the protruding edge), precisely where adhesions begin maturation ^57^. This indicates that actin retrograde flow is locally controlled by different mechanisms in the lamellipodium and the lamellum. One model proposes that the speed of the retrograde flow depends of the viscosity of the actin networks inside each subdomain ^58^. In this model, the lamellum exhibits a gel-like behavior (hence its mass flows rearwards slowly). Conversely, the lamellipodium behaves as a particulate liquid (hence it flows quickly, up to the gel-liquid interface). This model does not emerge from the apparent local packaging of the actin networks, which is similar or even higher in the lamellipodium as seen by electron microscopy; but it defines the viscosity of each region according to the size of the main population of individual actin filaments, which are small in the lamellipodium and large in the lamellum ^59^. The current state of the art implies that lamellipodial retrograde flow depends not only on the amount of actin polymerization and the local accumulation of actin regulators but also on the mechanical resistance opposed by the plasma membrane: the higher the mechanical resistance, the higher the retrograde flow, which is demonstrated by a local increase in the resistance of the plasma membrane ^58^.

In the lamellum, myosin II-driven actomyosin assemblies resist retrograde flow for a number of reasons: one is that the filaments are anchored actively to the adhesive foci, which are immobile with respect to the actin flow; also, they are much larger and more cross-linked than lamellipodial actin; thus, they present higher resistance against the dragging force of the retrograde flow; finally, they are further from the edge, where the mechanical resistance of the plasma membrane promotes the highest degree of retrograde flow. Other factors may contribute to the increased resistance in this region, such as the presence of large-scale cellular organelles (for example, the nucleus) and membrane-containing systems (for example, Golgi apparatus or the endoplasmic reticulum).

The adhesive clutch plays a key role in determining the fate of adhesive contacts. It is likely that lamellipodial nascent adhesions are decoupled from actin. In this region, actin flows at high speed ^60^, yet adhesions are stationary though short-lived ^8^. As the lamellipodium advances and adhesions are stationary, the lamellipodium-lamellum interface reaches them. Here, a decision is made, whether the adhesion disassembles or grows into a larger, elongated adhesion. Several studies have elucidated different requirements for elongation, including the actin cross-linker α-actinin and myosin II-A ^8, 61^. It is feasible that the increased actin bundling, combined with reduced actin speed, increases the degree of clutch engagement, enabling these adhesions to exert stronger overall traction on the substratum. This higher degree of clutch engagement is illustrated by the fact that, in this region, traction is directly related to the retrograde flow of actin ^57^, indicating that force transmission is more efficient.

## Integrin-based cell-cell adhesive complexes

Integrins not only ligate cells to the ECM but also can mediate cell-cell interactions, particularly transient ones. Most leukocytes express two major integrin receptors, α _L_β _2_ and α _4_β _1_, which are intrinsically inactive to prevent leukocyte adhesion to the blood vessel walls. Upon microbial attack, fibroblasts release tumor necrosis factor-alpha, which induces the endothelial expression of the major ligands for α _L_β _2_ and α _4_β _1_, intercellular adhesion molecule-1 (ICAM-1) and vascular cell adhesion molecule-1 (VCAM-1), respectively. This is, by itself, insufficient to trigger α _L_β _2_ and α _4_β _1_ activation. However, chemokines, which are released by fibroblasts and macrophages and also expressed on the surface of endothelial cells, drive the talin-dependent inside-out activation of α _L_β _2_ and α _4_β _1_. Integrins can then bind their ligands and promote the stabilization of endothelial cell-leukocyte contacts to permit extravasation toward the inflammatory site ^62^. A variation of the same model enables T lymphocyte recirculation through lymph nodes in homeostasis. Indeed, high endothelial venules leading into the lymph nodes constitutively express ICAM-1 and chemokines specific only for naïve T cells ^63^. Furthermore, circulating cancer cells adopt this model to extravasate in selected anatomical locations and promote metastasis ^64^.

Leukocytes are among the best-characterized models in which transient and inducible integrin activation occurs only in response to homeostatic alterations. However, integrin-based cell-cell contacts occur in many other scenarios, in which they mediate long-lived contacts (for example, between neurons forming synaptic contacts) (reviewed in 65).

## Toward an integrated model of dynamic cell-cell and cell-matrix adhesion to coordinate collective cell migration

**Figure 4.**
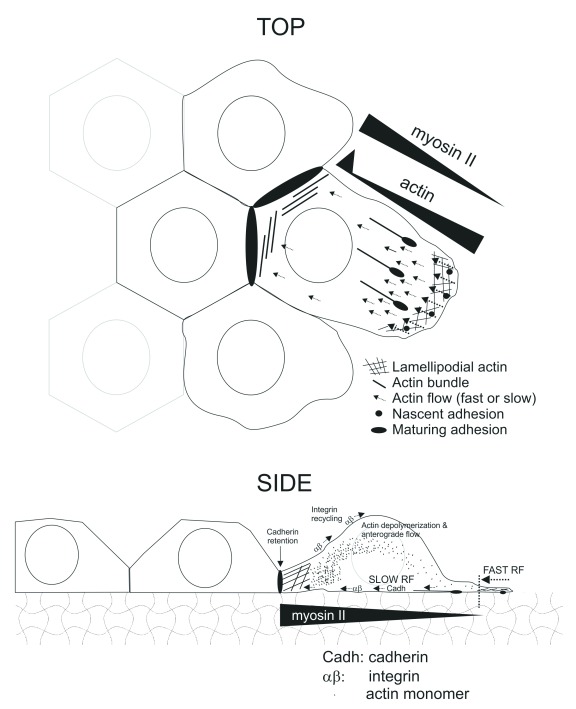
Integration of contact reinforcement and integrin-based clutching in the movement of leader cells. Top panel represents a top view of the scenario depicted in Figure 1. Additional details include the intensity of the retrograde flow (RF), which is strong in the lamellipodium and weak in the rest of the cell. Also represented are elongated adhesions associated to actin fibers as well as rearward accumulation of actin, myosin II, and cadherins. The gradients of actin and myosin II subcellular concentration are inverse, except at the boundary between leader and follower cells, where actin spikes again. Bottom panel represents a side view of the same situation, with RF close to the cell-substratum interface. The panel also represents integrin recycling to the front and actin monomer anterograde motion to replenish the polymerizable pool at the front of the cell.

Throughout this review, we have described adhesive complexes that mediate stable interactions between cells and other cells (or the ECM) as well as complexes that assemble and disassemble dynamically. How does it all fit together during coordinated phenomena (for example, collective cell migration)? Although the fine details of such events are largely unknown, some general principles have emerged. Cadherin-mediated cell-cell contacts maintain epithelial sheets together while enabling proliferation. Cells proliferate maximally, their growth limited by extracellular cues, which can be geometrical constraints or anti-proliferative barriers. Growth limitation can also be based on intracellular parameters (for example, cytoplasmic pressure and membrane strain). In these scenarios, mechanical parameters could modulate cadherin-driven signaling to favor, or halt, proliferation ^66^. In specific morphogenetic or homeostatic scenarios, migratory needs arise (for example, epiboly, dorsal closure, and wound repair) ^67, 68^. A common signal in these situations is the emergence of a migratory cue. The appearance of such a cue generates intrinsic asymmetry, with some cells closer to the cue. These cells develop motility-related features. These features may break down their contacts with the rest of the cell layer, turning these cells into solitary migrators. If the contact with the monolayer is not broken, these cells become “leaders” and propel the migration of the entire cohort toward the migratory cue. In this scenario, efficient coordination between cadherin- and integrin-dependent adhesive complexes is required for collective migration. Cadherin bonds between leader and follower cells become strained, undergoing force-dependent consolidation ( Figure 1 and Figure 4). At the same time, integrin-based cell-matrix adhesions form and turn over in a clutch-dependent manner in leader and follower cells alike ( Figure 3). The direct relationship between the dynamics of these adhesive contacts is unclear at present. A possible model of interplay includes multiple modes of cadherin reinforcement driven by changes in actin regimes due to integrin-mediated migratory adhesion. For example, the actin retrograde flow opposing forward motion in the leader cells may direct cadherins toward the rear of the leader cell, increasing the number of receptors at the leader/follower interface ( Figure 4). In support of this hypothesis, cadherin coupling to actin retrograde flow has been observed ^69^. This mechanism assumes that cadherins are retained at the cell-cell contact site, which can be mediated locally by lateral interactions between cadherins at this region. Likewise, the slow retrograde flow across the leader cell directs pre-formed filaments to the rear of the cell, increasing actin bundle availability at the contact between cells. In fact, a large accumulation of actin bundles is observed at the rear of migrating cells ( Figure 4). Although it is currently unclear whether cadherins can associate with preformed actin bundles, this would explain the rapid reinforcement of the contact. However, rearward actin accumulation could eventually limit protrusion by decreasing the amount of free actin monomers. A possible compensating event could be related to the accumulation of myosin II filaments at the rear of leader cells. Filaments with more myosin II would require less actin to exert a similar structural role at the rear. In this sense, migrating cells display actomyosin filaments of graded polarity, which bear more myosin in more posterior locations ^70, 71^. Also, myosin II may directly promote actin depolymerization ^72^. In addition to increasing actin monomer availability for anterior polymerization, actin depolymerization would release integrins from actin, promoting their anterograde recycling for the assembly of new adhesions as the leading edge advances ^73^ ( Figure 4).

At a mechanical level, the emergence of these asymmetric forces has to be balanced internally to preserve the integrity of the monolayer ^22^. Additional issues contribute to this process (for example, transcriptional changes that skew the cells to a more epithelial or a more mesenchymal migratory phenotype) ^74^. These changes influence the coherence of the monolayers as they advance, promoting, or preventing, the emergence of solitary migrators, which is of outstanding importance in cancer. Indeed, the acquisition of motile features by epithelial tumor cells is a hallmark of aggressiveness and has critical importance in the dissemination of solitary tumor migrators that can reach the bloodstream and migrate to distant locations to form secondary tumors.

## The new frontier: 3D adhesive interactions support individual and collective cell migration and shape whole tissues

Adhesion studies in 2D are limited by the fact that such a scenario rarely happens *in vivo*. However, their ease of use and the ability to extract biochemical, genetic and proteomic information of such samples make these studies important and revealing. However, some considerations need to be taken into account, particularly the following:

- In 2D, the Z aspect of the cell is intrinsically polarized, supporting adhesion at the bottom but leaving the cell unconstrained at the top. In three dimensions (3D), the Z dimension is not different from XY, except in some interface instances (for example, cells adhering to basement membrane layers, bone, and so on).

- Tractions in 2D environments are usually limited to the bottom XY plane, with the emergence of sophisticated contractile structures that use focal adhesions as anchorage sites. In 3D, actin fibers do appear but are usually less contractile than their 2D counterparts. Owing to feedback between traction and contraction, adhesions are smaller than in 2D ^75, 76^. Additional issues emerge from the use of matrix-coated glass surfaces, which are several orders of magnitude stiffer than any tissue in mammalian organisms except bone ^77^. The emergence of functionalized 2D hydrogels has alleviated this concern ^78^, although the intrinsic polarity issue is not resolved.

- The rheology of the tissue is a key factor in 3D. Three-dimensional lattices are networks made of fibers of different length, thickness, and packaging. These factors make them permissible for the migration of some cell types but impregnable for others. Packaging is a crucial factor, to the point that it enables the migration of some cells in the absence of integrin-mediated adhesion ^79, 80^. In these cases, friction of the plasma membrane against the matrix fibers creates sufficient traction to enable protrusion ^81^. In this context, membrane friction would engage a minimal, integrin-independent clutch that would permit forward membrane extension. This occurs in combination with the spontaneous generation of pressure differentials inside cells by geometrical constraining of the newly generated protrusion. The strangulation of a protrusion by the matrix fibers would create a pressure differential that would inflate the protrusion independent of lamellipodial actin ^82^. This seems to be the preferred mode of migration in stringent 3D environments with narrow pores.

- Some cell types engineer tunneling solutions to penetrate 3D matrices (for example, polarized metalloproteinase secretion) ^83^. In this case, the cells carve their own path, using proteolytic degradation to increase the pore size to enable their movement. Proteolysis combines with rearrangements of the matrix caused by integrin-dependent binding to enable cell movement and simultaneous rearrangement of the 3D lattice.

- In 3D, the nucleus becomes an active impediment for migration, whereas this is not the case in 2D. Different reports have indicated a crucial role for rearward myosin II in actively squeezing the nucleus forward in 3D lattices ^84^ and narrow gaps, both artificial ^50^ and cell-cell junctions ^85^. In contrast, the main function of myosin II in 2D is to generate XY traction at the cell-matrix interface ^86^, preserve the structural integrity of the cell ^87^, bring up the rear, and establish front-back polarity ^42^. It is worth mentioning that the nucleus has recently been proposed to act as an intracellular piston that generates intracellular pressure asymmetry that governs cell motility in confined spaces ^88^. Importantly, the role of the nuclear piston seems to be context-dependent, as highly proteolytic tumor cells do not use it unless matrix proteolysis is inhibited ^89^, suggesting a connection between matrix degradation (and possibly stiffness) and piston engagement.

These considerations are crucial during the shaping of tissues and organs. Cells deposit and remodel matrices, which determine the geometry and boundaries of the tissues, direct the morphological arrangement of the cells to form tissues with specific geometries, and maintain the homeostasis of these large-scale structures over extended periods of time.

## Concluding remarks

The vast amount of knowledge amassed over the last 40 years has yielded a detailed description of the molecular players involved in cell-cell and cell-matrix adhesions. However, their stoichiometry, dynamics, and especially their synergies and antagonisms are much less understood. This is a particularly critical aspect of cell biology as it impinges on deregulation mechanisms that potentially cause disease. Technical advances in quantitative imaging, proteomics, and epigenomics hold the key to reveal the intricate molecular interplay between cell-cell and cell-matrix adhesions to morph masses of cells into organized, fully functional tissues. At a molecular level, the clutch mechanism of integrin adhesion represents a crucial step in adhesive flexibility. It also cooperates with cadherin-based adhesions to control multicellular processes such as collective cell migration. The (relatively) novel discovery of the mechanical regulation of adhesive adaptors and their coupling to the actin cytoskeleton represent a tunable link in a continuous, mechanically active chain that connects extracellular properties to intracellular responses. These include morphological, transcriptional, and translational cell and tissue adaptations to the microenvironment. These conserved mechanisms represent a baseline level of complexity required for the specialization of tissues in multicellular organisms. However, increased complexity, as found in mammalian cells, resides in additional regulatory levels and molecules. These elements constitute a mechanical continuum, in which tissues and cells remain organized during organogenesis and regeneration and also ready to respond to external aggression or internal malfunction.
